# Planning, implementation, and sustaining high coverage of human papillomavirus (HPV) vaccination programs: What works in the context of low-resource countries?

**DOI:** 10.3389/fpubh.2023.1112981

**Published:** 2023-04-14

**Authors:** Dur-E-Nayab Waheed, Ana Bolio, Dominique Guillaume, Anissa Sidibe, Christopher Morgan, Emilie Karafillakis, Megan Holloway, Pierre Van Damme, Rupali Limaye, Alex Vorsters

**Affiliations:** ^1^Centre for the Evaluation of Vaccination, University of Antwerp, Antwerp, Belgium; ^2^Department of Infectious Disease Epidemiology, London School of Hygiene and Tropical Medicine, London, United Kingdom; ^3^Jhpiego, The Johns Hopkins University Affiliate, Baltimore, MD, United States; ^4^International Vaccine Access Center, Johns Hopkins Bloomberg School of Public Health, Baltimore, MD, United States; ^5^Center for Infectious Disease and Nursing Innovation, School of Nursing, Johns Hopkins University, Baltimore, MD, United States; ^6^Department of Vaccine Programmes, Gavi, the Vaccine Alliance, Geneva, Switzerland; ^7^Nossal Institute for Global Health, School of Population and Global Health, University of Melbourne, Melbourne, VIC, Australia; ^8^Department of International Health, Johns Hopkins Bloomberg School of Public Health, Baltimore, MD, United States; ^9^Department of Health, Behavior and Society, Johns Hopkins Bloomberg School of Public Health, Baltimore, MD, United States; ^10^Department of Epidemiology, Johns Hopkins Bloomberg School of Public Health, Baltimore, MD, United States

**Keywords:** HPV vaccination, low-and lower-middle-income countries, human papillomavirus, vaccine implementation, barriers and facilitating factors

## Abstract

Cervical cancer due to human papillomavirus (HPV) infection is a leading cause of mortality among women in low-resource settings. Many Sub-Saharan African countries have introduced HPV vaccination programs at the national level in the last few years. However, countries are struggling to maintain sustainable coverage. This study focuses on the introduction and sustainability challenges, context-specific key lessons learned, and mechanisms of action to achieve high sustainable coverage from low and lower-middle-income countries (LLMICs) that have introduced HPV vaccination programs by collating evidence from a literature review and key informant interviews. Local data availability was a challenge across countries, with the lack or absence of registries, data collection and reporting mechanisms. Multi-sectoral coordination and early involvement of key stakeholders were cited as an integral part of HPV programs and facilitators for sustainable coverage. Key informants identified periodic sensitization and training as critical due to high staff turnover. Health workforce mobilization was fundamental to ensure that the health workforce is aware of the disease etiology, eligibility requirements, and can dispel misinformation. Schools were reported to be an ideal sustainable platform for vaccination. However, this required teachers to be trained, which was often not considered in the programs. District-level staff were often poorly informed and lacked the technical and logistic capacity to support vaccination rounds and data collection. To improve the sustainability of HPV vaccination programs, there is a need for timely microplanning, efficient preparedness assessment, assessing training approaches, periodic training, finding innovative ways to achieve equity and adoption of a bottom-up approach to ensure that processes between districts and central level are well-connected and resources are distributed efficiently.

## 1. Introduction

Cervical cancer due to human papillomavirus (HPV) infection is a leading cause of morbidity and mortality among women in low-resource settings, accounting for approximately 88% of all cervical cancer deaths globally (1). Licensure of the first HPV vaccine in 2006 altered the landscape for cervical cancer prevention, with vaccination becoming a key component in prevention programs, introduced in approximately 130 countries to date (2).

Countries have employed various strategies in introducing HPV vaccines, some implemented pilot demonstration programs or phased roll-out prior to national introduction, while others immediately introduced HPV vaccines into their national immunization plan (3). HPV vaccination has been rolled out through different delivery platforms such as schools, health facilities, or community outreach. Schools are often the predominant primary delivery site in low-and lower-middle-income countries (LLMICs) targeting mainly 9–14 year-old girls as per WHO recommendations (4). As of mid-2021, 7 of 29 low-income countries (LICs), and 21 of 51 lower-middle-income countries (LMICs) had introduced HPV vaccine (2), initially correlated with the availability of financial support, e.g., Gavi, the Vaccine Alliance (2), but most national introductions in LLMICs started after 2017 (5) followed by the WHO call to elimination of cervical cancer globally in 2018 (6).

Since HPV vaccination programs are relatively new in LLMICs, national decision-makers have faced several introductory challenges in addition to the global shortage of HPV vaccine including selection of optimal delivery strategy, community level communication, targeting hard to reach populations (out of school girls, populations living with HIV infection, internally displaced peoples, refugees, ethnic minorities, etc.), tailoring delivery and communication approaches to sub-national level, and determining how to maximize coverage (7, 8). Recent introductions have highlighted the need for context-specific solutions to sustain introduction and delivery of HPV vaccination programs. Additionally, there is a growing body of evidence that the ongoing covid-19 pandemic has created additional challenges of vaccine prioritization and increased workload on health systems in the immunization domain (9).

Drawing on published data and key informant interviews (KIIs), this study aimed to summarize lessons learned from recent HPV vaccination program introductions, develop a deeper understanding of facilitators and barriers to HPV vaccination programs, and identify key areas for achieving sustainable coverage.

## 2. Methods

We conducted a qualitative, multi-method study design comprising a systematic literature review and semi-structured KIIs to determine key barriers and identify what works and why in HPV vaccination programs. After collating our results, we drew on concepts from theory-based evaluation to deepen our analysis of service delivery experiences in LLMICs by extracting and synthesizing findings on how contextual elements trigger specific barriers and mechanisms of action that could improve outcomes. The service delivery experience analysis was focused on HPV introduction and post-introduction experiences.

### 2.1. Systematic literature search and literature review

We performed a systematic literature search in August 2021 using a combination of different keywords, including individual names of LLMICs that had introduced or planned to introduce HPV vaccination programs (10). The detailed search strategy with keywords and MESH terms with Boolean terms is available in Supplementary Table S1.

**Figure 1:**
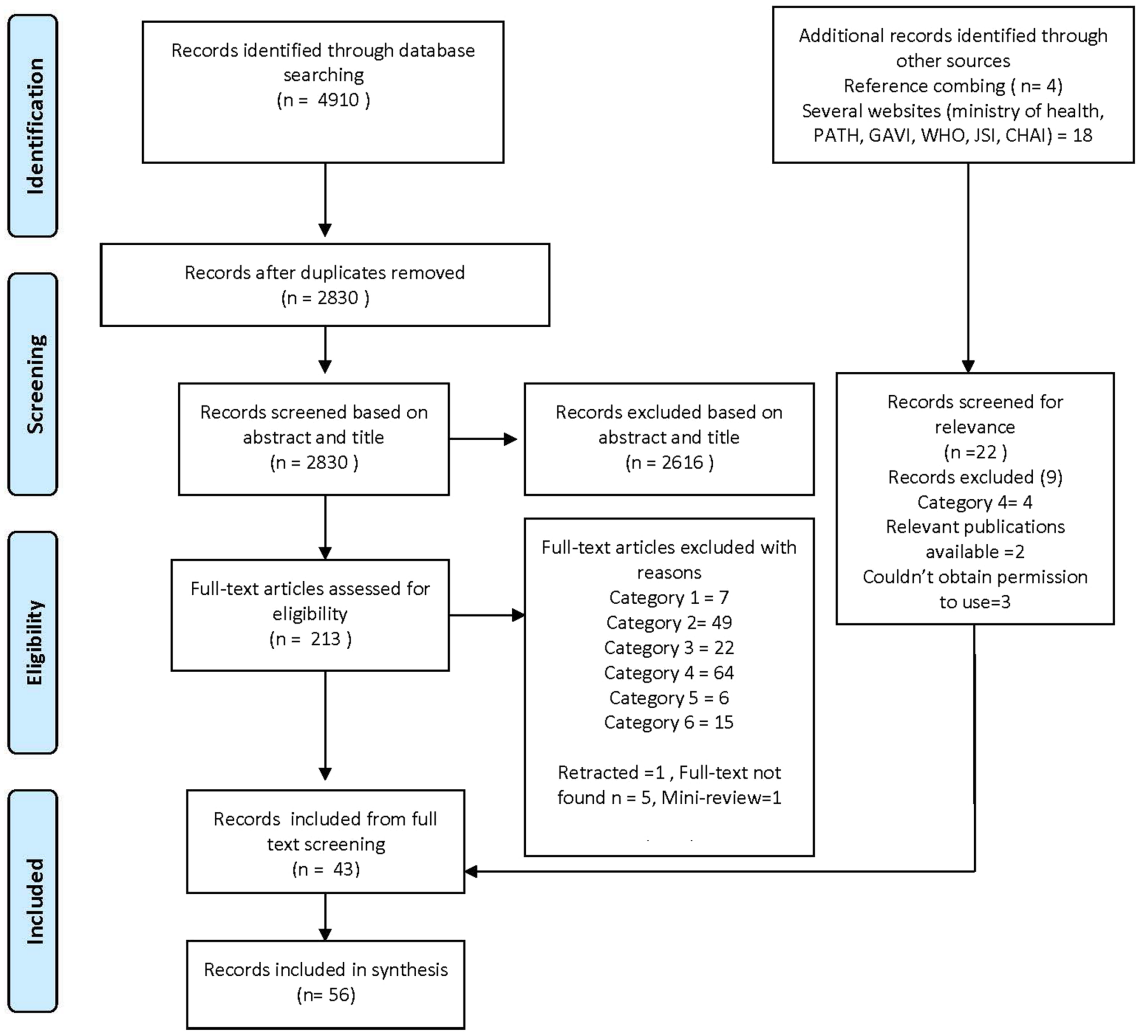
Systematic literature review flow. Publications in the second round were excluded if (1) from HICs and failed to be excluded in the first round, (2) from Upper middle-income countries (UMICs), (3) covered personal beliefs, knowledge, acceptability, uptake, vaccine hesitancy and attitudes among parents, women and adolescents, (4) not focused on the national HPV vaccination program, (5) hypothetical acceptance studies or studies discussing disease burden before vaccine introduction, and (6) detailed modeling studies on cost-effectiveness analysis or cost estimation of HPV vaccination programs.

Four databases were searched in a systematic way: Ovid, PubMed, Web of Science, and Science Direct. Of 4,910 publications retrieved, we discarded duplicates in Endnote and Covidence and shortlisted 2,844 articles for abstract/title screening. Two team members (AB and DNW) screened and shortlisted articles using a screening tool based on inclusion/exclusion criteria, leaving 213 articles for full-text screening as per PRISMA guidelines (Figure 1). A detailed protocol is available on Prospero (11). Authors conducted reference combing and systematically searched one database (ProQuest) and several websites (Ministries of Health (MOH), Gavi, WHO, John Snow, Inc. (JSI), Clinton Health Access Initiative, Inc. (CHAI)) for grey literature including presentations and reports from various HPV focused projects through August and Sept 2021 (12, 13).

Following full-text screening, we shortlisted 43 articles that included the following themes: decision-making, planning and implementation, social mobilization and communication, sustainability and lessons learned from HPV vaccination programs. To keep findings context-specific, we focused only on LLMICs.

### 2.2. Key informant interviews (KIIs)

Ten LLMICs based in Asia and Africa were identified purposefully to ensure diversity in lessons learned based on: (1) introduction status, i.e., introduced or planned introduction, (2) programmatic diversity, i.e., delivery strategy, dosing schedule, target age cohort etc., (3) vaccine coverage, and (4) geographical diversity. HPV-related publications, documents and country evaluation documents were used to prepare the initial national and sub-national level stakeholders’ list, and a snowball approach was used to identify additional experts. Key stakeholders were defined as members of the National Immunization Technical Advisory Group (NITAG), senior officials from national departments of health, and representatives from international organizations, e.g., WHO and the United Nations Children’s Fund (UNICEF). Ethical approval for the study was obtained, and participants were asked to provide an informed consent. We interviewed 18 national HPV experts from February 2022 to April 2022 (until saturation) *via* Zoom to gain insight into HPV vaccine introduction experiences and associated challenges in their respective countries (Table 1). Country identification for KIIs was independent of the studies identified in the review, which may overlap but were not deliberately kept the same. Although we aimed to maintain diversity in HPV vaccine introduction status across selected countries, we were not able to secure interviews from the countries planning HPV national vaccination programs. Therefore, the findings of KIIs are based on countries having a national HPV vaccination program.

**Table 1:** Overview of countries included in systematic literature search and stakeholder interviews.

1.1. Systematic literature search: Countries and publication included from literature review			1.2 Key informant interviews: Countries included and stakeholders interviewed	
Countries	Income status	Date of national introduction	Country	Expertise
Bhutan (8, 16–18)	LMIC	2009	**National level stakeholders (*n* = 10)**	
Cameroon (19)	LMIC	2020	Cote d’Ivoire	National immunization program and economics
Côte d’Ivoire	LMIC	2019	Ethiopia	National immunization program and public health
Ethiopia (20)	LIC	2018	Kenya	National immunization program
Kenya (20–22)	LMIC	2019	Lao PDR	National immunization program
Lao PDR (23)	LMIC	2020	Rwanda	National immunization program
Malawi (24–26)	LIC	2019	Zambia	National immunization program
*Mozambique* (27, 28)	LIC	2021	Zimbabwe	Health promotion and policy
Rwanda (16, 20, 29–32)	LIC	2011	**Non-governmental organizations (NGOs) (*n* = 8)**	
Senegal (5, 33)	LMIC	2018	Cote d’Ivoire	National immunization program
Tanzania (20, 34–39)	LMIC	2018	Kenya	Gynecology and public health
Uganda (16, 40–45)	LIC	2015	Lao PDR	National immunization program
Zambia (20)	LIC	2019	Myanmar	National immunization program
Zimbabwe (20, 46–49)	LMIC	2018	Zambia	National immunization program
LLMICs* (3, 7–9, 16, 50–55)	-	-	Zimbabwe	National immunization program

Section 1.1 shows countries included in the study with publications from the systematic literature review. Countries in italics are those that introduced during the review process and the study covers experience only from demonstration programs from the respective country.

* indicates publications that anonymously report findings, i.e., without indicating countries separately or summarized the findings of LLMICs. Lao PDR -Lao People’s Democratic Republic. The definition for LICs and LMICs is used from World Bank data; low-income economies are defined as those with a GNI *per capita*, calculated using the World Bank Atlas method, of $1,085 or less in 2021; lower middle-income economies are those with a GNI *per capita* between $1,086 and $4,255 (75). Section 1.2 illustrates the countries, stakeholder category and expertise of the stakeholders interviewed.

### 2.3. Data extraction and analysis

#### 2.3.1. The literature review

Two authors (DNW, AB) independently extracted data from the peer-reviewed and grey literature search from December 2021 –January 2022 using an Excel-based matrix comprising key themes informed by WHO guidelines for new vaccine introduction and introducing HPV vaccine into national immunization program guide (14, 15). The matrix was piloted *a-priori* and revised twice to allow for emerging themes and was reviewed independently by the third author (AV). The matrix included domains of decision-making, planning, social mobilization and communication, monitoring and evaluation, sustainability, associated challenges and lessons learned. Data were grouped by year of introduction, country, and primary delivery strategy to study different experiences and draw best practices.

We used the Joanna Briggs Institute Critical Appraisal checklist tools to evaluate the risk of bias in the articles included in the study.

#### 2.3.2. Key informant interviews

Transcripts of KIIs conducted on Zoom were iteratively analyzed by two researchers (DNW and DG) independently using thematic analysis with inductive and deductive coding developed *a-priori* from the semi-structured interview guide [S2]. Two authors randomly selected two transcripts and conducted open coding and after discussing any discordance and disagreements, developed a codebook to use to analyze the remaining transcripts using ATLAS.ti v22 (56). We used information obtained in the interviews to consolidate and document challenges reported in the literature, especially for countries that introduced HPV vaccination programs between 2019 and 2021. Study findings were synthesized in a narrative approach to discuss interviews and literature review themes that reinforced each other. Sub-themes that emerged from the interviews were data collection, management and reporting, and human resource capacity.

## 3. Results

We analyzed 43 peer-reviewed articles and 13 grey literature documents, the latter comprising country evaluation reports (*n* = 4), PATH country presentation documents (*n* = 2), JSI country reports (*n* = 2), country briefs (*n* = 2) and a viewpoint (*n* = 1), WHO Bulletin (*n* = 1) and CHAI resource document (*n* = 1) (Supplementary Table S4). Fourteen LICs and LMICs from Africa and Asia, 11 of which introduced national HPV vaccination programs between 2018–2021, are included in the study with publications from the systematic literature review (Table 1). Experience from eight national programs in Africa and Asia were covered in 18 interviews (Table 1).

We categorized results by drawing on the domains outlined in the WHO guide on introducing HPV vaccines in national immunization programs. We categorize results using the following themes: preparation and planning, implementation, social mobilization and communication. As we included countries with existing national HPV immunization programs in our analysis, we added two additional themes: sustainability, and impact of Covid-19 on HPV vaccination programs.

## 4. Preparation and planning

### 4.1. Local data availability, data reporting, and management

Health information systems such as databases or registries to record and report data on vaccination are essential for decision-making (57), optimal planning, tracking and determining accurate coverage. Lack of local service data was highlighted as a major challenge and contributed to delaying the decision-making process.

Paper based registries were the most common reporting tool used during vaccination sessions at schools and data registries were often not integrated with the routine immunization data collection. In some countries where reporting, data storage and tracking systems were not optimized, accessing data during school breaks and determining accuracy of collected data was a challenge (46). Key-informants reported that transferring paper-based data to electronic sheets and timely retrieval of data was a significant challenge (e.g., in Zambia).

Availability of data for enumerating eligible girls is also crucial for planning the vaccination sessions and was often inaccurate or inconsistent across sectors and programs. Countries have adopted various approaches in enumerating lists of in and out of school girls which often involved consolidating these lists from district to national level through various meetings (33, 47). For example, the presence of health extension posts facilitated enumerating out-of-school girls in Ethiopia. (KIIs).

Defining the target age cohort was critical to enumerate schoolgirls such as in Senegal and Zimbabwe where the target cohort was of an age that transitioned between grades and/or schools during the vaccination window. This complicated registration and tracking processes. Furthermore, access to registers at school during school breaks, accuracy of data collected, and discrepancies between records at schools and health facilities were reported as barriers for data reporting and management (33, 46).

### 4.2. Vaccine management: Vaccine supplies, cold chain, and other logistics

Global shortage of HPV vaccine was cited as a major challenge at the national level across all programs, prompting many countries (e.g., Ethiopia, Zambia, Tanzania) to switch from vaccinating multi-age cohorts to single-age cohorts (20) (KIIs). Numerous bottlenecks for vaccine management at sub-national levels were found in our study: insufficient vaccines on the day of administration (due to inaccurate quantification of numbers of eligible girls), challenges in transporting vaccine to the site and distant locations, inadequate facilities in healthcare centers such too few working fridges, highlighting the need to ensure adequate storage capacity during preparedness assessment and microplanning (40, 41, 48). Countries with limited local vaccine storage capacity at the district level faced challenges with vaccine management and supply during the vaccine administration days specifically for the campaign mode as large number of vaccines had to be administered during a short period of time. Findings showed that the most efficient form of cold chain management for HPV vaccination is incorporation with routine immunization infrastructure (8). Ethiopian key-informants voiced logistics as a significant challenge for program managers such as limited funds for staff transportation and *per diem* allowances for conducting vaccination sessions in schools.

Key informants emphasized previously reported staff shortage (42) for conducting HPV immunization activities, further exacerbated by staff relocation to tackle covid-19 pandemic-related activities across eight countries (9) (Table 1). Stakeholders also strongly voiced the need of strengthening staff technical capacity at national and district levels.

### 4.3. Multi-sectoral coordination

HPV vaccination programs require not only the involvement of coordinating bodies and the health workforce but also different governmental and non-governmental organizations to facilitate planning, introduction and implementation activities at the national, regional, and district levels. One critical partner is the Ministry of Education (MoE) (58), as LMICs deliver the HPV vaccine primarily through school outreach sessions.

Often, countries did not have a pre-existing adolescent health program at the time of HPV vaccine introduction; thus, lack of experience with nontraditional partners raised implementation challenges. However, Rwanda’s program experience demonstrated that early inclusion of the MoE, Ministry of Gender and Family Promotion, research institutes, health workforce, and additional stakeholders is possible even without an adolescent health intervention at the EPI level (29, 59). The experience of Cote d’Ivoire, where some schools did have school health programs but were not utilized in the initial planning, emphasizes the need for robust mapping and microplanning prior to introduction (KIIs). Similarly, various program experiences found inclusion of MoE and Ministry of Finance (MoF) in preparatory meetings, experience from past immunization programs, trust between stakeholders, local champions and political prioritization as critical factors for efficient coordination (43, 50).

Interview findings provided an in-depth understanding of the challenges to partner coordination; often the challenges started from the initial decision-making and planning processes. Lack of clarity on vaccine prioritization processes and competition for program placement among stakeholders, with some not involved or missed, created resentment and difficulties with resource mobilization and planning. Involvement of international stakeholders *via* different national stakeholders created lack of ownership. Barriers such as changes in staff and leadership (44), lack of efficient resource distribution from national to district levels (KII), under-representation of MoF (43), disconnect between new vaccination introduction and processes within the districts (49) are also critical to partner coordination (16) (27).

## 5. Implementation

### 5.1. Training

Stakeholders cited training as a critical factor to ensure that the health workforce and related stakeholders are fully aware of the vaccine, the disease etiology, target age cohort, delivery strategies, data collection and can mitigate misinformation. Duration and content of training (33, 34, 47) was similar across countries. To deliver training, countries used a combination of presentations, demonstrations, group work, separate training guides and role-plays, varying from 1 day (e.g., Zimbabwe, Senegal) to 3 days of training (e.g., Tanzania, where additional topics beyond vaccination were covered) (33, 35, 47). Cascade training was the most common training approach across programs (33, 35, 47). The most frequent challenges in this approach were high staff turnover, dilution of information in the cascade approach and extension of HPV training beyond the usual EPI target, adding to the need for training and tools to tackle misinformation. An alternative approach that worked well for Laos PDR was centralized team training where expert groups were sent to districts to minimize the dilution of training in the cascade training approach.

Another barrier to training was budget. To cut costs, training was often combined with other vaccination programs, conducted virtually, or packaged with non-vaccine interventions as in Tanzania (24, 40). However, in Cote d’Ivoire, this approach resulted in healthcare workers having limited knowledge and capacity to advocate for HPV vaccine in the field.

HPV training program needs extend beyond the usual EPI target age cohort adding to the need for content-focused interactive training approaches. Healthcare professionals (HCPs) are mainly responsible for vaccination, mobilization and communication activities, including responding to rumors about HPV vaccines. However, HCPs were sometimes found to have little knowledge of disease etiology and vaccine, with some reports indicating that HCPs were unaware of the disease they were vaccinating against or the MoH-recommended target age group eligible for vaccination. In addition, in Uganda and Tanzania, only a few HCPs reported attending a condensed one-day training vaccine a few weeks before the introduction (36, 41). In specific instances (such as Cote d’Ivoire) the planning process and trainings were rushed or missed.(KIIs) Similar lack of training and communication was observed in low performing districts in Uganda (45). Optimal training and involvement of teachers (60, 61) was identified as a facilitator for sustainable coverage. One of the emerging findings from our study was the critical need to focus on HCP training content and refresher training due to staff turnover. All key-informants strongly voiced the need for periodic training, as training was usually only anticipated for initial introduction with no planned refresher training or HCP capacity building.

### 5.2. Delivery

Countries need to select a delivery approach and primary delivery site for HPV vaccination depending on vaccine supply, feasibility of access to sites, distance between health facility and schools, school attendance and cost-effectiveness of each parameter. The two main alternatives in timing of HPV vaccine delivery approaches include campaign and regular routine. In routine delivery, vaccine is available all year round. In contrast, in campaign delivery, the vaccine is administered in campaigns lasting a few days, a week or month; usually at six-or twelve-month intervals.

In many countries, schools are the primary delivery site while a smaller number opted for healthcare facilities, depending on the ease of access to the target population. We analyzed different delivery approaches and the relevant facilitators and barriers per country (Table 2). Eleven of the national HPV vaccination programs in sub-Saharan LLMICs covered in our study have adopted schools as primary or secondary vaccination sites (Table 2). All programs supplemented delivery activities with either availability of the HPV vaccine at health facilities following vaccination sessions or through mobile posts or community outreach platforms. All six of the SEARO and WPRO region LLMICs opted for schools as primary or secondary delivery sites.

**Table 2:** Barriers and facilitators related to HPV vaccine delivery. The table includes barriers and facilitators related to HPV vaccine delivery including cross-cutting themes such as microplanning, communication and social mobilization.

	Countries	World bank income status	Introduction date	Primary delivery site	Secondary delivery site/outreach	Target age cohort & dosing schedule	Adolescent health program	Coverage		Key lessons learnt
								HPV1 (%)	HPVc (%)	
	*Delivery Approach -Campaign*									
1	Ethiopia	LIC	2018	School	–	SAC –14-year-old (oldest eligible girls) Dosing schedule: 0, 6	No	2019:		**Facilitators:** High school attendance Participation of regional education bureau in introduction activities; Close collaboration between MoH and MoE. **Barriers:** Limited funding for staff transportation and *per diem* allowance for vaccination sessions in school ^(KIIs)^.
								94	84	
								2020:		
								95	76	
								2021:		
								86	75	
2	Zimbabwe	LMIC	2018	School	Health facility –mop up for 1 month at HF after 1 week of school-based campaign	MAC -10 to 14-year-old girls (first year); grade 5 (subsequent years) dosing schedule: 0, 12	No	2019:		**Facilitators**: 12 months dosing schedule to harmonization the vaccine sessions with school calendar-continued MOH and MOE collaboration (49). **Barriers:** confusion on age eligibility among HCPS visiting schools creating missed opportunities (46, 47), accurate data reporting on day of vaccination, lack of messages on second dose for girls in the school.
								91	67	
								2020:		
								n/a	n/a	
								2021:		
								67	40	
3	Rwanda	LIC	2011	School	Health facility: 2 days school-based campaign followed by limited availability at the health facility	SAC –9-year-old Dosing schedule: 0, 6	No	2019:		**Facilitators:** Strong multi-sectoral coordination between MoE and MOH (29) Training and involvement of school teachers ^[KII]^.
								97	94	
								2020:		
								89	68	
								2021:		
								78	73	
4	Zambia	LIC	2019	School	Health facility School as primary delivery site integrated into child health week	SAC –14-year-old Dosing schedule: 0, 12	No	2019:		**Facilitators:** the initial schedule was 6 months for two doses, switched to 12 months to align with the school calendar Integration with child health days. **Barriers:** lack of clear communication during child health days on administration of HPV vaccine ^(KIIs)^.
								99	n/a	
								2020:		
								75	69	
								2021:		
								45	33	
5	Lao PDR	LMIC	2020	Community based	Initially school but changed to community outreach due to Covid-19	MAC -10-to 14 year-old girls (1st year); 10-year-old girls (subsequent years) Dosing schedule: 0, 12	No	2020:		**Facilitators:** Central training approach that facilitated trained HCPs on the field to conduct vaccination ^[KII]^.
								76	n/a	
								2021:		
								37	42	
	*Delivery Mode –Routine*									
6	Gambia		2019	School	Out of school girls visit schools on vaccination days	SAC –9-year-old Dosing schedule: 0.12	No	2019:		No information could be retrieved
								68	n/a	
								2020:		
								n/a	n/a	
								2021:		
								34	30	
7	Malawi	LIC	2019	Static and outreach clinics –vaccine available throughout the year as each girl turns 9	Schools (quarterly delivery in schools)	SAC –9-year-old Dosing schedule: 0, 6	No	2019:		Switch from campaign to routine mode in 2021. **Facilitators**: Health surveillance assistance (HSAs), a committed team focused on conducting vaccine related activities including communicating on the importance of vaccination tackling misinformation, mobilization community and school staff (25). **Barriers:** inadequate funding to train all health workers and teachers for delivery (24).
								88	n/a	
								2020:		
								84	77	
								2021:		
								14	12	
8	Tanzania	LMIC	2018	School	Community sites, community mobile sites, health facility	SAC –14-year-old Dosing schedule: 0, 6	No	2019:		**Facilitators:** Periodic sensitization and distribution of flyers to increase coverage (35). **Barriers:** Inadequate engagement between health facilities and schools Outreach activities were not integrated into health programs. Lack of precise messages on the second dose in schools (36).
								78	49	
								2020:		
								82	58	
								2021:		
								73	57	
9	Uganda	LIC	2015	School	Health facility and outreach posts	SAC –10-year-old 0, 6 months	No	2019:		**Facilitators:** Integration with child health days. **Barriers:** unclear communication on the target cohort to HCPs resulting in some turning down the eligible girls, transport challenges and limited vaccines for vaccination sessions in schools.
								99	64	
								2020:		
								74	30	
								2021:		
								75	44	
10	Cote d’Ivoire	LMIC	2019	School	Health facility – supplemented by community outreach	SAC –9-year-old Dosing schedule: 0, 6	No	2019:		**Facilitators:** Periodic intensification of routine immunization –focused on the school-based campaign –as catch-up during covid-19. Covid-19 prompted specific innovative messaging to encourage girls to visit health facilities during school closure.
								6	n/a	
								2020:		
								67	13	
								2021:		
								34	41	
11	Kenya	LMIC	2019	Health facility	Advocacy, communication and social mobilization support through communities schools	SAC – 10-year-old Dosing schedule: 0, 6	No	2019:		**Barriers:** Vaccine misinformation among community members, Lack of involvement of religious groups planning and introduction activities affecting delivery and consequently coverage.
								25	n/a	
								2020:		
								33	16	
								2021:		
								29	44	
12	Senegal	LMIC	2018	Health facility	Outreach at community locations and primary schools – frequency determined by HCF	SAC – 9-year-old Dosing schedule: 0, 6	No	2019:		**Facilitators:** Close collaboration b/w MoE and MoH -incorporated a module on primary cervical cancer prevention in school curriculum, planning sessions in collaboration with school. 1 day training -developed separate training guides for teachers and HCPs (33). **Barriers:** Not all schools in districts had trained teachers and not all facilities received HPV communication material.
								86	25	
								2020:		
								45	31	
								2021:		
								39	21	

The coverage data is obtained from the IVAC view hub and HPV dashboard (10, 76). Countries with data retrieved for the study are included in the table. ‘n/a’ refers to countries that introduced 1st dose in 2019 or 2020, and did not have dose 2 coverage yet per reporting year. HPV1 (%) refers to HPV vaccine first dose coverage and HPVc (%) refers to HPV vaccine second dose coverage. Low- income countries (LIC). Lower-middle-income country (LMIC). Single-age cohort (SAC). Multi-age cohort (MAC).

In terms of different delivery approaches, Ethiopia, Zimbabwe, Rwanda (30), and Zambia opted for the campaign delivery approach with annual school campaigns ranging from 2 days to 1 week. Although considered expensive, stakeholders appreciated a campaign mode harmonized with the school calendar with fewer focused visits of healthcare teams as an effective approach. An additional facilitating factor was harmonizing the dosing schedule with school calendars. Integrating the HPV vaccination campaign in child health weeks or similar scheduled events (as in Zambia) was a facilitator for campaigns. Countries opting for campaign delivery need to be prepared to mobilize resources, intensify communication, and periodically sensitize staff. Table 2 shows 2019 and 2020 coverage rates in countries opting for campaign or routine delivery approaches, campaign showing higher coverage coupled with schools as primary delivery platform compared with routine approach. (60, 61). Routine mode might work better for countries with sub-optimal school attendance with intensified social mobilization and communication outreach activities. These program experiences have demonstrated that providing vaccination at school is both feasible and acceptable (20, 36, 62). Using a school-based vaccine delivery platform with a six-month or annual dose schedule is an effective strategy, but it requires careful micro-planning between health and education officials (17, 46), and at times the information forwarded from MoH to school administration did not reach the school teachers (63) (KIIs). There is insufficient data on programs using health facilities as the only site for vaccine delivery.

Integration of HPV vaccination and adolescent health programs in schools was recommended by WHO as a promising practice (64). However, none of the countries discussed in this review had a strong pre-existing adolescent or school health program ready for the addition of a new intervention like vaccination. This challenged coordination and microplanning as new partners such as MoE needed to be engaged to support school outreach visits. For instance, in Cote d’Ivoire, initially the MoE was seen as simply a healthcare service delivery site; it was not until sometime after introduction that more relevant education leaders with a mandate to provide a school health program were identified and enlisted in-depth support for HPV vaccination. Despite these limitations, countries have shown that high school attendance, timely inclusion and coordination with MoE, a committed health workforce, training and mobilization of school staff can enable HPV vaccination delivery.

#### 5.2.1. Healthcare workforce

Healthcare workers are crucial for an efficient delivery strategy provided the vaccination sessions logistics are well planned. Recent literature reports that lack of remuneration for healthcare workers in Uganda resulted in demotivated or unsatisfied staff which disrupted delivery (41). A critical but underappreciated consideration was the need for the extra time and resources needed to staff in school outreach visits. Similarly, key-informants reported that teachers who supported vaccination activities felt left out if compensation was offered to their counterparts (i.e., healthcare workers) on the day of vaccination.

#### 5.2.2. Microplanning at the district level

Microplanning is necessary to ensure that national and district level processes are well prepared, connected, and sustainable (44), with a district tailored delivery approach. This includes determining number of vaccines required, sessions in schools, and assessing resource capacity at each level, among others, well before introduction (16, 49). Several HPV vaccination programs, including Ethiopia and Zambia, mentioned having conducted microplanning specifically to target each age cohort and out-of-school girls. Schools were asked to develop lists of girls eligible for vaccination, and that information was sent to healthcare facilities. However, it was sometimes challenging for countries to implement activities outlined in micro-plans. Some challenges reported were unpredictable vaccination sessions (Uganda (41)), enumerating the correct target population due to unavailability of local data (Malawi (24)), and unclear communication between MoE, MoH and school management. In Lao PDR, an additional challenge was that the communication material was sent to the district level without any instructions. Funding constraints for HPV vaccine introduction limited the ability to carry out all activities for communication, introduction and monitoring as laid out in the micro-plan (27).

## 6. Social mobilization and communication

Social mobilization and communication for HPV vaccination require active engagement and commitment beyond the usual coordinating bodies. A variety of key stakeholders and communications channels are needed, given the target age group and the need for prior sensitization within communities and professional organizations. It also requires a well thought-out plan and funding for sustainability beyond the initial introduction. Some countries (Tanzania, Zimbabwe, Senegal, Zambia, Kenya) had a communication plan in place with experts from local and international organizations to reach different target audiences across national, regional and district levels. Communication plans included guidelines for advocacy and sensitization meetings, mass media involvement, launching ceremonies, identification of target groups, and the activities and materials meant for each target group as well as a crisis management plan. Printed products containing key messages were developed and distributed to schools, communities, and healthcare facilities through mass and social media. Senegal incorporated a module on cervical cancer in the school curriculum with collaboration between MoE and MoH (33).

Only few publications in the systematic review provided detailed data on social mobilization and communication in countries. While these experiences intensively discussed the structure of social mobilization and communication plans prior to introduction, very few programs reported on the timing and duration of these activities.

In terms of message content, the Ugandan experience demonstrated that simple messages to girls, especially regarding vaccine safety and efficacy, are useful for vaccine uptake. Similarly, the need for periodic sensitization and circulation of communication material was emphasized by KIIs (34, 36).

Senegal and Zimbabwe conducted radio broadcasts for communication and social mobilization activities which proved to be very useful in remote areas with limited or no access to television, and communication was tailored to local languages. Kenya supplemented these activities with talk shows and mass social media outreach (21). Novel approaches to enhance communication have been reported in Cote d’Ivoire and Senegal where WhatsApp groups have been used to communicate effectively between program managers and HCPs to tackle emerging HPV vaccine misinformation.

Not all the planned communication activities translated into high uptake of HPV vaccine. Therefore, to develop an in-depth understanding through consolidating literature review findings and KIIs, some critical facilitators included the need for early mobilization of stakeholders at all levels (31), involving political and religious leaders and journalists in national and regional stakeholder meetings to timely address the rumors about HPV vaccine affecting fertility, and sufficient funding to conduct periodic sensitization activities. Key-informants reported limited supply of social mobilization and communication materials (e.g., flyers and posters) as a challenge (e.g., in Lao PDR) and in-depth program evaluation reported that communication material was sent to district level without any instructions.

## 7. Sustainability

Sustainability is commonly conceptualized as the ability to sustain high coverage within a smoothly running program, and, where programs are mature enough to enable this, to integrate HPV vaccination with other adolescent health programs. Our findings suggest further that, in some cases, sustainability also requires strengthening existing infrastructure for vaccine management and capacity building of the healthcare workforce to ensure a committed workforce (65) and increase engagement with early adolescence to achieve optimal coverage (66). Some review findings (e.g., Tanzania) have shown that post introduction support, such as supportive supervision after vaccination rounds, can be beneficial to maintain engagement and momentum among the healthcare workforce (34, 35). Tanzania plans to implement an HPV vaccine recovery effort to fill the gap in communication activities and to increase collaboration with MoE (35). Rwanda has demonstrated that co-delivering HPV vaccination with other health interventions in schools is possible (67). However, the overall literature review findings lack data on potential integration with broader adolescent health services or enabling landscape for investment in adolescent health. All key-informants reported not having a plan at the national level for integration with existing or potential health adolescent health intervention.

Transitioning out of Gavi support often presents LLMICs with a challenge, threatening sustainability if the country cannot sustain finances for running a stable HPV vaccination program (KIIs). In such scenarios, lessons learned from other countries, such as Bhutan, that collaborated with partners for long term sustainability planning are critical (18).

## 8. Covid-19 pandemic and hpv vaccination programs

The covid-19 pandemic disrupted immunization activities widely but more so for HPV vaccination programs as most countries deliver HPV primarily through schools which still remain closed (April 2022) in some countries (e.g., Lao PDR). Key-informants indicated that HPV vaccination programs have very low priority across programs, compounded by parents being hesitant to bring their daughters to healthcare facilities (e.g., in Cote d’Ivoire) especially during the covid-19 pandemic, since it was difficult to conduct outreach activities. Hence, Cote d’Ivoire adopted a Periodic Intensification Routine Immunization (PIRI) approach and switched to campaign mode to increase coverage and use available vaccine supply. There is an increasing need for a consolidated contingency plan to ensure the continuation of HPV vaccination as with other routine immunization services. Programs such as Myanmar that launched during covid-19 had to switch to a community-based delivery approach instead of a planned school-based delivery approach. Zambia reported that integrating HPV vaccination in child health week allowed minimal sustainability of the program compared with other country experiences during the covid-19 pandemic (KIIs). The coverage from year 2021 is included in Table 2 to demonstrate impact and not to correlate with delivery approaches. The drop in coverage across all countries calls for consolidated back-up plan for delivery yet requires in-depth case studies to learn from resilient examples such as Ethiopia and Rwanda.

Covid-19 overstretched the system leaving less room for HPV-related activities in low-resource countries, including resources diverted from routine immunization services to covid-19 and overworked healthcare workforces. Similarly, small private health facilities were closed in some countries, adding further pressure to overstretched immunization activities (21).

## 9. Discussion

**Figure 2:**
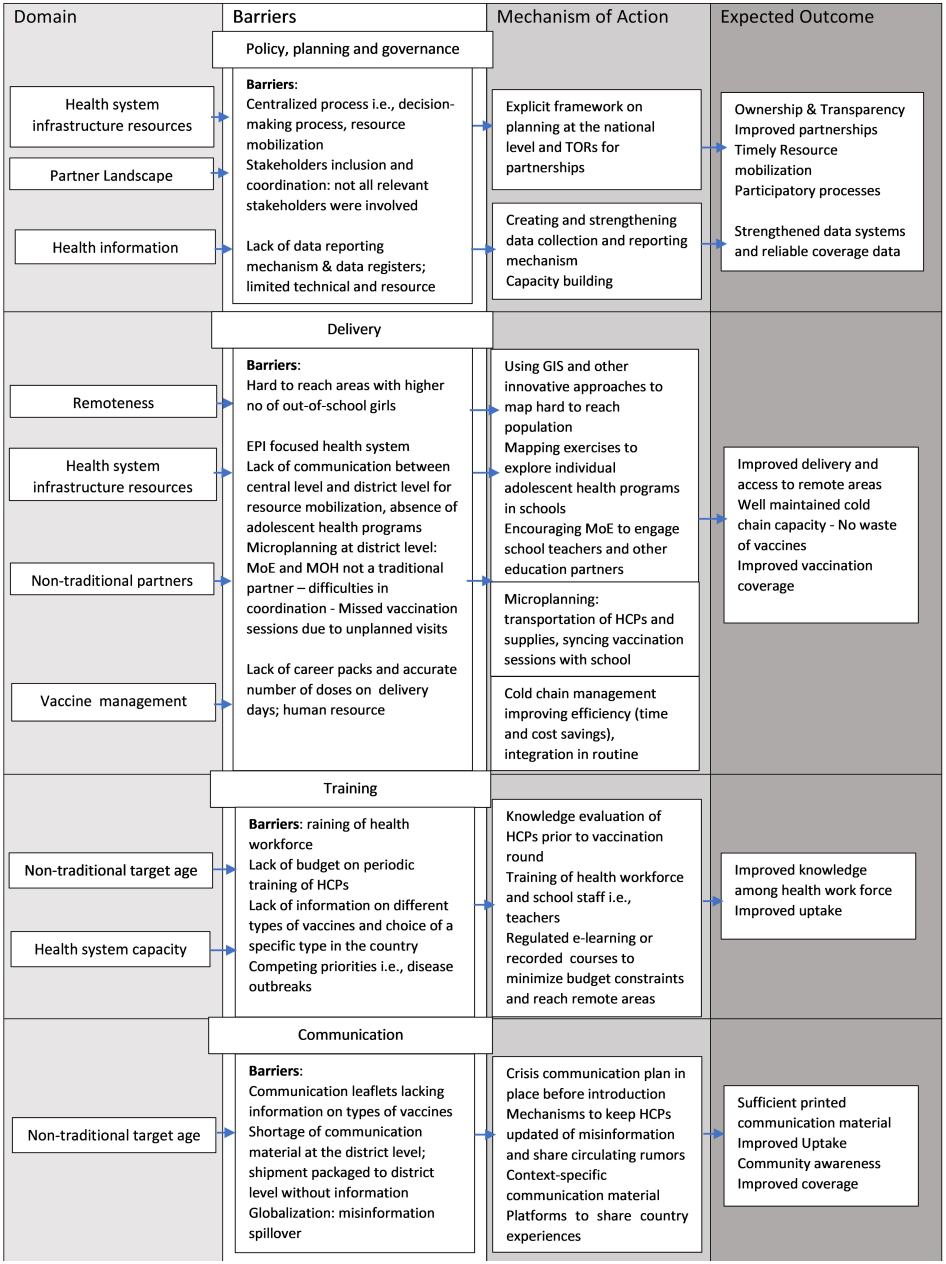
Domain-Specific Barriers and Mechanisms of Action for Enhancing HPV Vaccination Program Performance. This figure includes domain-specific barriers to various parameters of HPV vaccination programs. These barriers can be addressed through mechanisms of action that can improve outcomes for the HPV vaccination program.

Successful elements of pre-introduction planning and implementation must work in concert to achieve sustainable HPV vaccination programs. To capture these interactions in our synthesis of findings, we considered how success factors worked together in different domains, overcoming barriers discussed in results through different mechanisms, to achieve sustainable program outcomes (Figure 2).

Planning requires reliable local data, efficient vaccine management and strong multi-sectoral coordination, While not explicitly found in our results, it also requires adequate time, sensitization including creating awareness among parents and adolescents (5). HPV programs must overcome considerable challenges in coordination between partnerships and program placement as demonstrated by our study and others, (35, 68, 69) necessitating stakeholder mobilization well in advance specifically for non-traditional partners such as MoE, professional associations, community and religious leaders. Explicit frameworks for planning can aid in overcoming such challenges. Figure 2 discusses domain-specific barriers which can be addressed with a set of actions to help achieve a sustainable HPV vaccination program. One potential mechanism of action suggested by our study to address barriers related to multi-sectoral coordination is to have a prioritization framework outlining vaccine prioritization processes and promoting use of terms of reference to outline the composition, roles, and responsibilities of different stakeholders, developing and sustaining collaboration between institutions that have not worked together (16), defining how the health workforce collaborates with teachers and community leaders during vaccination sessions (27), and promoting a strong national ownership framework.

LLMICs in Africa have struggled to accurately estimate doses and assess storage space. In countries with centralized equipment management coupled with lack of preparedness assessment results in challenges with cold chain capacity and insufficient vaccine storage packs, this could be improved by timely preparedness assessment and controlled temperature chain (Figure 2) (70). Timely cold chain management and microplanning down to facility level would help reduce loss of vaccines and attain higher coverage. As identified in a 2018 review (3), it is critical to increase attention on residual implementation barriers and conduct research on context-specific delivery approaches, as outlined in Figure 2. There is no one-size-fits all approach for sustainable high coverage based on the delivery strategy. Delivery requires advance microplanning which may vary depending on the delivery platform that is utilized. For instance, if vaccines are to be delivered within school-based platforms there is a need to harmonize vaccination sessions with school calendars as well as improve registers and understanding target population (19). The approach should be local (71), based on strengthened stakeholder coordination, cost-effectiveness, and feasibility analyses of delivery approaches that work well down to the district level as logistics and school enrolment are not homogenous across a country.

All key informants reported lack of or inadequate training of HCPs as a challenge. Training is critical component and as countries experience ongoing competing priorities, emerging misinformation and budget constraints, it is imperative to adequately budget, evaluate and adopt innovative strategies, conduct healthcare workforce mobilization, followed by training for HPV vaccination and invest in capacity building of healthcare workforce (24). Trainings should extend beyond the conventional guides, involve school staff and include updated information on vaccine types (i.e., why a specific type is administered in the country) and how to mitigate misinformation. Communication challenges are common globally; misinformation spill-over from HICs to LLMICs and circulating rumors were found to be critical barriers in our study and others (72). The use of digital communication technologies, as we reported in Cote d’Ivoire and Senegal, can improve coordination among managers and be a more potent tool to tackle misinformation. Increasing knowledge among HCPs and reducing misinformation is needed for successful scale-up of HPV vaccination, as HCPs are typically considered the most trustworthy source of information on health services and can significant influence on vaccine acceptance and uptake (73, 74).

Recently, the WHO Strategic Advisory Group of Experts on Immunization (SAGE) concluded that a single dose of HPV vaccine is comparable to two-dose schedules in protecting against cervical cancer (77). Switching to one-dose HPV vaccination will open the opportunity to vaccinate girls, particularly within remote areas, without loss to follow-up, which tends to be a significant barrier against high coverage. Table 2 shows countries can achieve higher first dose coverage. However, it is important to highlight that while one dose may increase coverage, it still requires strengthening planning and implementation activities (i.e., cold chain capacity, enumerating girls to be vaccinated, local data availability for decision-making, strengthening delivery platforms, communication, periodic training due to staff turnover, strengthening data collection and reporting systems). There is a need for investment from governments to strengthen human resources, infrastructure capacity such as cold chain management and distribution requirements (65), capacity building of healthcare workforce, robust cost-analysis tools, new implementation partners and increased coordinated global investment (51).

## 10. Limitations

This study excluded publications and topics relating to data costing models, or potential cost-effectiveness that focused only on evidence to support vaccine introduction decisions. We did not include papers that focused on vaccine sentiment or hesitancy (given the breadth of that field) if they did not address issues related to planning or implementation of HPV vaccine programs. Using this criteria enabled us to focus on themes related to planning and implementation once the vaccine was introduced, and allowed us to have an in-depth discussion on key parameters of HPV vaccination programs. Similarly, the authors focused on LLMICs to glean context-specific lessons learned. The authors relied on information generated from peer-reviewed literature, available grey literature, and KIIs with a limited and purposefully selected group of country stakeholders; therefore, the authors acknowledge that some information could have been missed. The KIIs covered HPV stakeholders at the national level, with findings covering eight LLMICs in Africa and Asia.

## 11. Conclusion

Our study documented the gaps in data collection and reporting mechanisms, the importance of multi-sectoral coordination, and the need to proactively engage stakeholders (such as the Ministry of Education) with a defined framework. Furthermore, it covered HPV delivery and training approaches, highlighting the need for context-specific implementation research through a series of recommendation in Table 3 based on mechanism of action discussed in Figure 2 to achieve sustainable coverage. Key informants reported the need for coordinated investment for periodic training of the health workforce, contextualized messages for communication and innovative approaches for hard-to-reach target populations. It is imperative to work toward improving partnerships among stakeholders, efficient health workforce mobilization and capacity building, research on sustainable delivery approaches, strengthening health information systems, and centering the voices of local stakeholders to achieve sustainable HPV vaccination programs.

**Table 3:** Recommendations for the sustainable HPV vaccination program.

HPV Vaccination program parameters	Recommendations for improving program coverage
Planning	Preparedness assessment before introduction and adoption of a bottom-up approach for microplanning, drawing on multiple population data sources. Allowing sufficient time for planning before the introduction. Need to develop platforms for local data collection and reporting. Capacity Building –providing on-job training to the healthcare workforce and updating them about the latest advancements in the field. Close collaboration between MoE and MOH. Widening landscape of implementation partners.
Training	Core training group to prevent dilution of training at the core level. Need for robust mechanisms to counter misinformation and equip HCPs with sufficient information and communication tools. Resource allocation to training teachers.
Delivery	Efficient microplanning based on good local intelligence on student numbers, locations and behaviors. Using combinations of delivery strategies to bridge gaps in remote areas and for out-of-school girls. Identifying and enlisting organizations outside of the health system who also have a mandate to improve student health, including education and adolescent health stakeholders. Building meaningful roles for education staff rather than just using schools as clinic outreach points. Accurate resource mobilization to ensure sufficient logistics and human resource capacity to vaccinate girls either at school or health facility.
Communications	Comprehensive message framing includes the types of vaccines and a specific type that is administered in the country. Communication plan that understands and leverages existing strengths and trusted focal points in local context. Digital technologies for rapid coordinated communications to build confidence and respond to misinformation. plan. Sustaining consistent communications beyond the introduction phase. Early preparation and the involvement of multiple stakeholders in community sensitization (media, religious, and political leaders). Messages should focus on cervical cancer prevention, safety and efficacy, including no impact on fertility.
Social mobilization	Social mobilization should begin at least 2 months prior to the introduction. Engaging important stakeholders at an early stage Stakeholder mobilization, including involvement of certain key stakeholders in the planning and microplanning process to initiate ownership in the implementation process. Periodic social mobilization until the program matures. Consistent social mobilization and community engagement activities before and during the vaccination to maintain high-level demand among the community.
Sustainability	Need for periodic training/refresher training due to staff turnover. Communication contingency plan must be embedded in the introduction Need for a holistic approach; strategies to integrate HPV as a routine practice like other childhood vaccines. Robust post-introduction evaluation visits to provide assistance and motivate the health workforce. Strengthening and encouraging local research institutes to generate local data. Crisis plan in place to ensure sustainability of HPV vaccine delivery platforms.

## Data availability statement

The original contributions presented in the study are included in the article/Supplementary material, further inquiries can be directed to the corresponding author.

## Ethics statement

The studies involving human participants were reviewed and approved by The Ethics committee of Antwerp University Hospital. The patients/participants provided their informed consent to participate in this study.

## Author contributions

DNW, CM, EK, and AV worked on the study’s protocol. DNW, AB, and AV conducted the systematic literature review. DNW and DG conducted the qualitative key-informant interview study. DNW: conceptualization, writing original draft, reviewing, and editing. AV: conceptualization, reviewing, and editing. AB, AS, CM, EK, DG, MH, RL, and PVD –reviewing and editing. All authors contributed to the article and approved the submitted version.

## Funding

The key-informant interviews study was supported by funding from the Bill and Melinda Gates Foundation under INV-006006 (PI: Rupali Limaye, JHU). The funders had no role in the design and conduct of the study nor the decision to prepare and submit the manuscript for publication.

## Conflict of interest

The authors declare that the research was conducted in the absence of any commercial or financial relationships that could be construed as a potential conflict of interest.

## Publisher’s note

All claims expressed in this article are solely those of the authors and do not necessarily represent those of their affiliated organizations, or those of the publisher, the editors and the reviewers. Any product that may be evaluated in this article, or claim that may be made by its manufacturer, is not guaranteed or endorsed by the publisher.
