# Supplementary material for: Planning, implementation, and sustaining high coverage of human papillomavirus (HPV) vaccination programs: What works in the context of low-resource countries?
Source: Front Public Health. 2023 Apr 14;11:1112981. doi: 10.3389/fpubh.2023.1112981 (PMC10140426; doi:10.3389/fpubh.2023.1112981)
Supplement: Supplementary file 1 [file Data_Sheet_1.pdf]

Supplementary Material

Planning, implementation and sustaining high coverage of human papillomavirus (HPV) vaccination programs: what works in the context of low-resource countries?

Dur-e-Nayab Waheed*[1], Ana Bolio[2], Dominique Guillaume[3,4,5], Anissa Sidibe[6], Christopher Morgan[3,7], Emilie Karafillakis[1,2], Megan Holloway[6], Pierre Van Damme[1], Rupali Limaye[3,8,9,10] , Alex Vorsters[1]

*** Correspondence:** Corresponding Author: nayab.waheed@uantwerpen.be

S1 Table: Systematic search performed with combination of keywords and countries names used and results retrieved in the database: Ovid MEDLINE(R) ALL <1946 to August 03, 2021> - updated on August 04, 2021

1	HPV.af.	45914
2	HPV.mp.	45669
3	exp papillomavirus vaccines/	8713
4	papillomavirus.mp.	51967
5	1 or 2 or 3 or 4	60228
6	exp Immunization/	185598
7	Immunization Programs/ or Vaccination/ or Vaccin*.mp. or Vaccines/	409375
8	6 or 7	472354
9	Developing Countries/	77339
10	low-income countr*.ti,ab.	7429
11	middle-income countr*.ti,ab.	23070
12	(low and middle income countr*).ti,ab.	21219
13	9 or 10 or 11 or 12	99151
14	5 and 8 and 13	414
15	(Benin or Argentina or Burundi or Cameroon or Belize or Botswana or Brazil or Bulgaria or Cabo Verde or Cote d'Ivoire or Ethiopia or Gambia or Colombia or Cook Islands or Costa Rica or Kenya or Lesotho or Liberia or Dominica or Dominican Republic or Ecuador or El Salvador or Eswatini or Malawi or Fiji or Mauritania or Mozambique or Grenada or Guatemala or Nigeria or Rwanda or Sao Tome or Senegal or Sierra Leone or Jamaica or Tanzania or Togo or Uganda or Zambia or Zimbabwe or Bolivia or Libya or Guyana or Malaysia or Maldives or Honduras or Marshall Islands or Mexico or Micronesia or Montenegro or Morocco or Namibia or Armenia or North Macedonia or Georgia or Paraguay or Peru or Philippines or Moldova or Saint Lucia or Saint Vincent or Samoa or Uzbekistan or Bhutan or South Africa or Indonesia or Suriname or Myanmar or Thailand or Sri Lanka or Timor-Leste or Tonga or Turkmenistan or Tuvalu or Cambodia or Lao or Vanuatu or Mongolia or Solomon Islands).ab,ti.	472949
16	5 and 8 and 15	1088
17	14 or 16	1427
18	Limit 17 to last 10 years	1144

Understanding the HPV vaccine introduction landscape
PIs Name: Alex Vorsters, PhD & Rupali Limaye, PhD
In-Depth Interview Guide – English/French

S2: Key Informant Interview Guide: Country-Level Stakeholders

Thank you for taking time to speak with us today. This interview aims to better understand the decision-making processes that take place among key stakeholders (NGO, MOH) in the introduction and implementation of HPV vaccination programs in LMICs. Data obtained from this research will be used to support policy decision-making for HPV vaccine implementation moving forward.

The information you provide will be combined with information from other people and relevant documents to develop reports, manuscripts, and inform future workshops. In any external publications, we won't use your name, your designation, or your organization; however, we may refer to your region (e.g., West Africa or South Asia) or your country.

NATIONAL STAKEHOLDERS

Prioritization and decision-making

Let's first talk about how cervical cancer prevention and HPV vaccination are prioritized relative to public health and vaccination programs within LMICs.

1. Tell me about yourself - role/responsibilities, org, etc.

2. How is HPV vaccination structured in your country?
   Probes: delivery strategy (campaign mode or routine), primary and secondary delivery site (delivery site), target age cohort

3. Briefly, what are/were the biggest obstacles facing HPV introduction and scale-up in your country?

   a. Probes: supply, political commitment, demand issues, COVID

4. Why do you think these obstacles exist? What are the reasons for these obstacles?

5. How does the vaccine prioritization and decision-making process operate in your country at the national and sub-national levels?
   a. What are the current competing priorities for ministries of health and/or finance?
6. How was the HPV vaccine prioritized in the country?
-
   a. Which factors are considered in the prioritization of HPV vaccination given competing public health priorities?
   b. Probes: disease burden, importance of the health problem, efficacy, financial, health system capacity, etc.

7. Which **stakeholders** at national and sub-national levels are involved in decision-making processes about HPV introduction and **scale-up?**

   a. Are some stakeholders missing from these processes?

8. What are the challenges facing these **stakeholders** related to vaccine decision-making? Why do you think these challenges exist? What are the reasons for these challenges?

9. What is being done and by whom to address challenges to HPV introduction and **scale-up** at national level?

10. What are the challenges at sub-national level in decision-making process? (This question will be for sub-national level stakeholders)
    a. To what extent are sub-national stakeholders involved in the decision-making process?
    b. What gaps do you see at the sub-national level? Which national and sub-national stakeholders could fill these gaps?

11. How could international organizations support governments, NITAGs, professional associations, etc., to fill these gaps?
    a) What type of technical support might be helpful to support this decision-making process?
    b) Is there specific data that might be needed to support this decision-making process?
    c) Is there advocacy or communications work that might be needed to support this decision-making process?

12. Multiple data points (e.g. local disease burden and epidemiologic factors, vaccine efficacy and safety, cost efficacy data) are used to guide the decision-making process for the introduction of HPV vaccines. Can you describe the data that is typically prioritized in decision-making?
    a. Is local data readily available to guide decision-making?
    b. What data is used when local data is unavailable?
    c. How programmatic considerations (such as vaccine choice, dosing schedule etc.) are evaluated in decision-making? Is this sufficient?

13. Member expertise and diversity among key actors is critical in facilitating decision-making. In smaller countries with limited numbers of experts, have there been challenges in identifying key actors from certain professional backgrounds?
    a. How does this affect the functional capacity of advisory groups (e.g. NITAGs)? How are these challenges addressed?

Vaccine Delivery and Acceptance

The next few questions will focus on HPV vaccine delivery along with public acceptability of HPV vaccines and how these influence the decision-making process.

14. Are demand and communications issues given sufficient consideration in decision-making and planning?
    a. How is the ACSM (advocacy, communication and social mobilisation) plan structured?
    b. How could this be improved?
    c. At what stage of the decision-making process is assessing public acceptance toward the HPV vaccination considered?

15. How are health care workers trained for HPV vaccine delivery?
    What is being done to keep them updated with the latest information and to deal with misinformation and rumours?
    Probes: training sessions, duration and structure of training sessions, plan for training refreshers

16. Is vaccine acceptance or hesitancy a major issue influencing HPV vaccine introduction and **scale-up** in your country?

17. How are such challenges you've described being addressed and by whom?
    a. Probes: e.g., increasing education, mass communication and media campaigns to heighten awareness, community engagement)

16. HPV vaccination programs are often packaged with other adolescent health services to enhance vaccine delivery (e.g. GAVI Alliance integrating age-relevant services such as deworming and nutritional supplements, integrating HPV vaccination with sexual and reproductive health education).
    b. Is integration of HPV vaccination with other adolescent health services considered in the decision-making process in your country (countries that have introduced HPV vaccination programs)? or will be considered (countries that are planning to introduce) If yes, how are packages decided upon?
    b. What are the reasons that integration of HPV vaccination programs with other adolescent health services are considered/not considered?

Scale up of HPV Vaccination During the COVID-19 Pandemic

We would like to ask a few questions about the impact of COVID-19 on ***the scale-up*** *of HPV vaccination.*

17. How has COVID-19 impacted HPV vaccine introduction and scale-up in LMICs or your country? How will this play out over the next 12 months?

a. Describe how the COVID-19 pandemic has affected strategies for the delivery of HPV vaccines (e.g. school closures affecting HPV vaccine delivery)?

18. How has HPV vaccination remained a priority for high-burden LMICs in the context of the COVID-19 pandemic?
    a. Has the prioritization of HPV vaccines (and other vaccines) been diverted to COVID-19 vaccines during the pandemic?

    b. Are there any additional factors that now are considered in HPV vaccine introduction which were not a concern prior to the COVID-19 pandemic?

19. Has public vaccine hesitancy or mistrust related to the HPV vaccine increased during the COVID-19 pandemic?

Other

20. Are there any other issues about HPV prioritization or decision-making process that you think it would be important for us to know?

21. Are there other people you would recommend we speak to about these issues?

Abbreviations

HPV; Human Papillomavirus, NGO; Non-Governmental Organisation, MOH; Ministry of Health, ACSM (Advocacy, Communication, Social Mobilisation)

Supplementary Material – Critical Appraisal Tables

S3	Critical Appraisal Tables
Table S3.1	Critical appraisal results of included qualitative studies
Table S3.2	Critical appraisal results of included cross-sectional studies
Table S3.3	Critical appraisal results of included Text, Opinion and Commentary studies
Table S3.4	Critical appraisal results of included Systematic Review/Review studies
Table S3.5	Critical appraisal results included Mixed-Methods studies

Table S3.1 Critical appraisal results of included qualitative studies

**Citation**	**Q1**	**Q2**	**Q3**	**Q4**	**Q5**	**Q6**	**Q7**	**Q8**	**Q9**	**Q10**
Rujumba et al., 2021	Y	Y	Y	Y	Y	Y	U	Y	Y	Y
Mphuru, et al., 2021	Y	Y	Y	Y	Y	U	N	Y	Y	Y
Kucheba et al., 2021	Y	Y	Y	Y	Y	Y	N	U	Y	Y
Soi, et al.2020	Y	Y	Y	Y	Y	U	N	Y	Y	Y
Keehn et al 2020	Y	Y	Y	Y	Y	Y	U	Y	Y	Y
Wallace, 2017	Y	Y	Y	Y	Y	N	N	Y	Y	Y
Ghallagher et al., 2017	U	Y	Y	Y	Y	N	N	Y	U	Y
Ghallagher et al., 2017	U	Y	Y	Y	Y	N	N	Y	U	Y
Kabakama et al., 2016	Y	Y	Y	Y	Y	U	N	Y	Y	Y
Watson-Jones et al., 2015	U	Y	Y	Y	Y	U	N	Y	Y	Y
Mugisha et al., 2015	U	Y	Y	Y	Y	N	N	Y	Y	Y
MacPhail et al., 2013	U	Y	Y	Y	Y	N	N	Y	Y	Y

Y=Yes, N=No, U=Unclear, NA=Not Applicable

JBI Critical Appraisal Tool for Qualitative Studies - Q1. Is there congruity between the stated philosophical perspective and the research methodology? Q2. Is there congruity between the research methodology and the research question or objectives? Q3. Is there congruity between the research methodology and the methods used to collect data? Q4. Is there congruity between the research methodology and the representation and analysis of data? Q5. Is there congruity between the research methodology and the interpretation of results? Q6. Is there a statement locating the researcher culturally or theoretically? Q7. Is the influence of the researcher on the research, and vice-versa, addressed? Q8. Are participants, and their voices, adequately represented? Q9. Is the research ethical according to current criteria or, for recent studies, and is there evidence of ethical approval by an appropriate body? Q10. Do the conclusions drawn in the research report flow from the analysis or interpretation, of the data?

Table S3.2: Critical appraisal results of included cross-sectional studies

Citation	Q1	Q2	Q3	Q4	Q5	Q6	Q7	Q8
Li et al., 2021	Y	Y	NA	NA	N	N	Y	Y
LaMontagne et al., 2021	Y	Y	Y	Y	N	N	Y	Y
Garon et al., 2021	Y	Y	Y	Y	N	N	Y	Y
Nabirye et al., 2020	Y	Y	Y	Y	Y	Y	Y	Y
Garon et al., 2019	Y	Y	Y	Y	N	N	Y	Y
Ladner et al., 2014	Y	Y	Y	Y	U	U	Y	Y
Msyamboza et al., 2017	Y	Y	Y	Y	U	N	Y	Y
Ogembo et al., 2014	Y	Y	Y	Y	U	N	Y	Y
Ladner et al., 2012	Y	Y	Y	Y	N	N	Y	Y

Y=Yes, N=No, U=Unclear, NA=Not Applicable

JBI Critical Appraisal Tool for Cross-sectional studies – Q1. Were the criteria for inclusion in the sample clearly defined? Q2. Were the study subjects and the setting described in detail? Q3. Was the exposure measured in a valid and reliable way? Q4. Were objective, standard criteria used for the measurement of the condition? Q5. Were confounding factors identified? Q6. Were strategies to deal with confounding factors stated? Q7. Were the outcomes measured in a valid and reliable way? Q8. Was appropriate statistical analysis used?

Table S3.3: Critical appraisal results of included Text, Opinion and Commentary studies

**Citation**	**Q1**	**Q2**	**Q3**	**Q4**	**Q5**	**Q6**
Li et al., 2021	Y	Y	Y	Y	Y	NA
Toh et al 2021	U	U	No	Y	Y	Y
Jennings et al., 2018	Y	Y	Y	Y	Y	N
Bonner et al., 2018	Y	Y	Y	Y	Y	N
Howard et al., 2017	Y	Y	Y	Y	Y	NA
Kumar et al., 2021	Y	Y	Y	Y	Y	N
Soi et al., 2018	Y	Y	Y	Y	Y	N
Gallagher et al 2018	Y	Y	Y	U	Y	N
Dorji et al 2015	Y	Y	Y	Y	Y	Y

Y=Yes, N=No, U=Unclear, NA=Not Applicable

JBI Critical Appraisal Tool for Commentary, Text and Opinion studies - Q1. Is the source of the opinion clearly identified? Q2. Does the source of opinion have standing in the field of expertise? Q3. Are the interests of the relevant population the central focus of the opinion? Q4. Is the stated position the result of an analytical process, and is there logic in the opinion expressed? Q5. Is there reference to the extant literature? Q6. Is any incongruence with the literature/sources logically defended?

Table S3.4: Critical appraisal results of included Systematic Review/Review studies

Citation	Q1	Q2	Q3	Q4	Q5	Q6	Q7	Q8	Q9	Q10	Q11
Tsu et al., 2021	Y	N	U	Y	N	N	N	N	N	Y	Y
Kramer, 2021	Y	N	U	Y	U	N	N	U	N	Y	Y
Black et al., 2018	Y	N	N	N	U	U	N	N	N	Y	Y
Howard et al., 2017	Y	U	Y	Y	U	N	N	N	N	Y	Y
LaMontagne et al., 2017	Y	N	U	Y	NA	N	N	U	N	Y	Y
Tsu et al., 2014	Y	Y	Y	Y	Y	U	U	Y	N	Y	Y
Wigle et al., 2013	Y	Y	Y	Y	U	N	N	Y	N	Y	Y
Sankaranarayanan et al., 2013	U	N	U	Y	N	N	U	U	N	Y	Y

Y=Yes, N=No, U=Unclear, NA=Not Applicable

JBI Critical Appraisal Tool for systematic review studies – Q1. Is the review question clearly and explicitly stated? Q2. Where the inclusion criteria appropriate for the review question? Q3. Was the search strategy appropriate? Q4. Were the sources and resources used to search for studies adequate? Q5. Were the criteria for appraising studies appropriate? Q6. Was critical appraisal conducted by two or more reviewers independently? Q7. Were there methods to minimise errors in data extraction? Q8. Were the methods used to combine studies appropriate? Q9. Was the likelihood of publication bias assessed? Q10. Were recommendations for policy and/or practice supported by the reported data? Q11. Were the specific directives for new research appropriate?

Table S3.5: Critical appraisal results included Mixed-Methods studies

Citation	S		Q1					Q2					Q3					Q4					Q5				
	1	2	1.1	1.2	1.3	1.4	1.5	2.1	2.2	2.3	2.4	2.5	3.1	3.2	3.3	3.4	3.5	4.1	4.2	4.3	4.4	4.5	5.1	5.2	5.3	5.4	5.5
Abdullahi et al., 2020	Y	Y	Y	Y	Y	Y	Y	NA	NA	NA	NA	NA	NA	NA	NA	NA	NA	Y	Y	U	U	U	Y	Y	U	N	U
Kisaakye et al., 2018	Y	Y	Y	Y	Y	Y	Y	NA	NA	NA	NA	NA	NA	NA	NA	NA	NA	Y	Y	Y	Y	Y	Y	Y	Y	Y	Y
Kamya et al., 2017	Y	Y	Y	Y	Y	Y	Y	NA	NA	NA	NA	NA	NA	NA	NA	NA	NA	Y	Y	Y	U	Y	Y	Y	Y	Y	Y
Torres-Rueda et al., 2016	Y	Y	Y	Y	Y	Y	Y	NA	NA	NA	NA	NA	NA	NA	NA	NA	NA	Y	Y	Y	U	U	Y	Y	Y	U	Y
Ladner et al., 2016	Y	Y	Y	Y	Y	Y	Y	NA	NA	NA	NA	NA	NA	NA	NA	NA	NA	Y	Y	Y	U	U	Y	Y	Y	Y	Y

Y=Yes, N=No, U=Unclear, NA=Not Applicable

McGill Mixed Methods Appraisal Tool (MMAT) Version 2018 – S1. Are there clear research questions? S2. Do the collected data allow to address the research questions? Q1.1. Is the qualitative approach appropriate to answer the research question? Q1.2. Are the qualitative data collection methods adequate to address the research question? Q1.3. Are the findings adequately derived from the data? Q1.4. Is the interpretation of results sufficiently substantiated by data? Q1.5. Is there coherence between qualitative data sources, collection, analysis and interpretation? Q2.1. Is randomization appropriately performed? Q2.2. Are the groups comparable at baseline? Q2.3. Are there complete outcome data? Q2.4. Are outcome assessors blinded to the intervention provided? Q2.5 Did the participants adhere to the assigned intervention? Q3.1. Are the participants representative of the target population? Q3.2. Are measurements appropriate regarding both the outcome and intervention (or exposure)? Q3.3. Are there complete outcome data? Q3.4. Are the confounders accounted for in the design and analysis? Q3.5. During the study period, is the intervention administered (or exposure occurred) as intended? Q5.1. Is there an adequate rationale for using a mixed methods design to address the research question? Q5.2. Are the different components of the study effectively integrated to answer the research question? Q5.3. Are the outputs of the integration of qualitative and quantitative components adequately interpreted? Q5.4. Are divergences and inconsistencies between quantitative and qualitative results adequately addressed? Q5.5. Do the different components of the study adhere to the quality criteria of each tradition of the methods involved?

Table S4: Details of grey literature document identified in literature search.

Document Type	Country	Year	Published by
Country report	Kenya	2021	JSI [1]
Brief	Malawi	2021	JSI [2]
Brief	Tanzania	2020	JSI [3]
Viewpoint	Malawi	2021	JSI [4]
Country report	Zimbabwe	2018	JSI [5]
Presentation	LMICs	2021	PATH [6]
HPV evaluation country slides	Zimbabwe	2021	CDC Foundation, Centers for Disease Control and Prevention with support from Gavi[7]
HPV evaluation country slides	Senegal	2021	CDC Foundation, Centers for Disease Control and Prevention with support from Gavi[8]
HPV evaluation country slides	Tanzania	2021	CDC Foundation, Centers for Disease Control and Prevention with support from Gavi[9]
Presentation	LMICs	2016	PATH – LSHTM [10]
Resource document	Uganda	2918	CHAI[11]
WHO News Bulletin	Lao PDR	2020	WHO[12]
Country report	Rwanda	2013	Torres Rueda et al 2013 [13]

LSHTM: London School of Hygiene and Tropical Medicine. JSI: John Snow, Inc. CHAI: Clinton Health Access Initiative.

References:

1. JSI. NEW VACCINE, NEW COHORT, AND COVID-19 INTERRUPTIONS: Kenya's HPV Vaccine Introduction. 2021. Available from: https://www.jsi.com/resource/kenyas-hpv-introduction-and-jsis-experiences/?filters=W3sicGxvY2F0aW9uIjoid29ybGR3aWRlIn0seyJwc3ViX2xvY2F0aW9uIjoia2VueWEifSx7InBleHBlcnRpc2UiOiJpbW11bml6YXRpb24ifSx7InBhdXRvbG9hZCI6MX1d. Accessed on 8 September 2021.
2. JSI. HPV VACCINATION IN MALAWI: Lessons Learned from JSI's Experience Supporting Vaccine Introduction and Routinization. 2021. Available from: https://www.jsi.com/resource/hpv-vaccination-in-malawi-lessons-learned-from-jsis-experience-supporting-vaccine-introduction-and-routinization/. Accessed on 8 September 2021.
3. JSI. Introducing Human Papillomavirus Vaccine In Tanzania 2020. Available from: https://www.jsi.com/resource/introducing-human-papillomavirus-vaccine-in-tanzania/. Accessed on 8 September 2021.
4. Patrick Nicks HH, Kate Bagshaw, and Nicole Davis. Community Health Workers: The Key to HPV Vaccination Success With Adolescent Girls in Malawi2021. Available from: https://www.jsi.com/community-health-workers-the-key-to-hpv-vaccination-success-with-adolescent-girls-in-malawi/.Accessed on 8 September 2021.
5. JSI, Ministry of Health and Child Care and the Department of the Zimbabwe Expanded Program on Immunization. LESSONS LEARNED: HPV VACCINE NATIONWIDE INTRODUCTION IN ZIMBABWE. 2018. Available from: https://www.jsi.com/resource/lessons-learned-hpv-vaccine-nationwide-introduction-in-zimbabwe/. Accessed on 08 August 2021.
6. Scott LaMontagne EM. National Introduction of HPV Vaccination in Low and MiddleIncome Countries: Lessons Learned from Formal Post Introduction Evaluations. 2021. Available from: https://path.azureedge.net/media/documents/HPV_PIE_Review_Slides_Final_PATHwebsite_2021Sep21.pdf. Accessed on 08 August 2021.
7. CDC Foundation, Centers for Disease Control and Prevention . Zimbabwe HPV Vaccine National Introduction HPV evaluation country slides 2021. Available from: https://www.dropbox.com/sh/kf2vt1ktrw33fim/AADVPQjdsNAkDvHn1Fl84c89a?dl=0&preview=Zimbabwe_Part+1AND2_FINAL_2.12.21.pdf. Accessed on 8 August 2021.
8. CDC Foundation, Centers for Disease Control and Prevention. SENEGAL HPV VACCINE NATIONAL INTRODUCTION - COUNTRY CASE STUDY & LESSONS LEARNED. 2021.Available from: https://www.dropbox.com/sh/kf2vt1ktrw33fim/AADVPQjdsNAkDvHn1Fl84c89a?dl=0&preview=Senegal_Part1AND2_FINAL_2.12.21.pdf. Accessed on 08 August 2021.
9. CDC Foundation, Centers for Disease Control and Prevention. Tanzania HPV Vaccine National Introduction. Country evaluation slides 2021.Available from:https://www.dropbox.com/sh/kf2vt1ktrw33fim/AADVPQjdsNAkDvHn1Fl84c89a?dl=0&preview=Tanzania_Part1AND2_FINAL_2.9.21.pdf. Accessed on 8 August 2021.
10. London School of Hygiene and Tropical Medicine P. Implementing HPV Vaccination: A review of seven keythemes for decision-makers. 2016.Available from: https://path.azureedge.net/media/documents/PATH-LSHTM_HPVvacll_slides_2016.pdf. Accessed on 08 August 2021.

11. Snidal S. Lessons learned from Uganda: Delivering HPV vaccines to hard-toreach girls2018 08/09/2021. Available from: https://www.clintonhealthaccess.org/lessons-learned-uganda-delivering-hpv-vaccines-hard-reach-girls/. Accessed in September 2021.
12. Lao PDR started nationwide HPV vaccination. Global Immunization News. World Health Organization 2020 [Available from: https://cdn.who.int/media/docs/default-source/immunization/gin/gin-june-2020.pdf.
13. Sergio Torres Rueda SR, Sandra Mounier-Jack., Helen Burchett, Maurice Gatera. Assessment of the impact of introducing the HPV vaccine on the immunisation programme and health system in Rwanda. Supplementary Report 2013.
